# Circulating Receptor Activator of Nuclear Factor kB Ligand and triglycerides are associated with progression of lower limb arterial calcification in type 2 diabetes: a prospective, observational cohort study

**DOI:** 10.1186/s12933-020-01122-4

**Published:** 2020-09-18

**Authors:** Olivier Bourron, Franck Phan, Mamadou Hassimiou Diallo, David Hajage, Carole-Elodie Aubert, Aurélie Carlier, Joe-Elie Salem, Christian Funck-Brentano, Salim Kemel, Philippe Cluzel, Alban Redheuil, Jean-Michel Davaine, Ziad Massy, Romuald Mentaverri, Dominique Bonnefont-Rousselot, Philippe Gillery, Stéphane Jaisson, Cees Vermeer, Jean-Marc Lacorte, Nesrine Bouziri, Suzanne Laroche, Chloé Amouyal, Agnes Hartemann

**Affiliations:** 1 Sorbonne Université, Paris, France; 2 Assistance Publique-Hôpitaux de Paris (APHP), Diabetology Department, La Pitié Salpêtrière-Charles Foix University Hospital, Paris, France; 3 Unité de Recherche Clinique Salpêtrière - Charles Foix, AP-HP, Hôpitaux Universitaires Pitié Salpêtrière - Charles Foix, 75013 Paris, France; 4 Department of Pharmacology and CIC-1421, AP-HP La Pitié Salpêtrière Charles Foix University Hospital, Paris, France; 5 INSERM, CIC-1901, Paris, France; 6 Institute of Cardiometabolism and Nutrition ICAN, Paris, France; 7 Laboratoire d’Imagerie Biomédicale INSERM_1146, CNRS_7371, Paris, France; 8 Assistance Publique-Hôpitaux de Paris (APHP), Department of Radiology, La Pitié Salpêtrière-Charles Foix University Hospital, Paris, France; 9 Division of Nephrology, Ambroise Paré Hospital, AP-HP, Université Paris-Saclay, Paris, France; 10 INSERM_1088, Centre Universitaire de Recherche en Santé, Université de Picardie Jules Verne, Amiens, France; 11 Department of Metabolic Biochemistry, La Pitié Salpêtrière-Charles Foix University Hospital (AP-HP), Paris, France; 12 UTCBS, CNRS UMR8258 – INSERM_1267, Faculty of Pharmacy of Paris, University of Paris, Paris, France; 13 University of Reims- Champagne-Ardenne, CNRS, MEDyC UMR 7369, Reims, France; 14 Laboratory of Biochemisry-Pharmacology-Toxicology, University Hospital of Reims, Maison Blanche Hospital, Reims, France; 15 Cardiovascular Research Institute CARIM, Maastricht University, Maastricht, The Netherlands; 16 Department of Endocrine and Oncologic Biochemistry, AP-HP, Pitié-Salpêtrière Hospital, Paris, France; 17 INSERM U1166, Paris, France; 18 INSERM, UMR_S 1138, Centre de Recherche des Cordeliers, Paris 06, France; 19 Département de Santé, Centre de Pharmacoépidémiologie (Cephepi), CIC-1421, Sorbonne Université, INSERM, Institut Pierre Louis d’Epidémiologie et de Santé Publique, AP-HP, Hôpitaux Universitaires Pitié Salpêtrière-Charles Foix, 75013 Paris, France; 20 Sorbonne University, ACTION Study Group, INSERM, UMRS 1166, Institut de Cardiologie, Hôpital Pitié-Salpêtrière (AP-HP), Paris, France; 21 Diabetology Department, Pitié-Salpêtrière Hospital, 47-83 Boulevard de l’Hôpital, Paris, France

**Keywords:** Arterial calcification, Biomarkers, Calcium score, Mediacalcosis, Peripheral artery disease, Type 2 diabetes

## Abstract

**Background:**

Lower limb arterial calcification is a frequent, underestimated but serious complication of diabetes. The DIACART study is a prospective cohort study designed to evaluate the determinants of the progression of lower limb arterial calcification in 198 patients with type 2 diabetes.

**Methods:**

Lower limb arterial calcification scores were determined by computed tomography at baseline and after a mean follow up of 31.20 ± 3.86 months. Serum RANKL (Receptor Activator of Nuclear factor kB Ligand) and bone remodeling, inflammatory and metabolic parameters were measured at baseline. The predictive effect of these markers on calcification progression was analyzed by a multivariate linear regression model.

**Results:**

At baseline, mean ± SD and median lower limb arterial calcification scores were, 2364 ± 5613 and 527 respectively and at the end of the study, 3739 ± 6886 and 1355 respectively. Using multivariate analysis, the progression of lower limb arterial log calcification score was found to be associated with (β coefficient [slope], 95% CI, p-value) baseline log(calcification score) (1.02, 1.00–1.04, p < 0.001), triglycerides (0.11, 0.03–0.20, p = 0.007), log(RANKL) (0.07, 0.02–0.11, p = 0.016), previous ischemic cardiomyopathy (0.36, 0.15–0.57, p = 0.001), statin use (0.39, 0.06–0.72, p = 0.023) and duration of follow up (0.04, 0.01–0.06, p = 0.004).

**Conclusion:**

In patients with type 2 diabetes, lower limb arterial calcification is frequent and can progress rapidly. Circulating RANKL and triglycerides are independently associated with this progression. These results open new therapeutic perspectives in peripheral diabetic calcifying arteriopathy.

*Trial registration* NCT02431234

## Background

Arterial calcification is particularly prevalent in ageing, diabetes or renal failure [1]. Lower limb arterial calcification is observed in up to 50% patients with type 2 diabetes and is independently associated with cardiovascular morbidity, renal failure and mortality [2–4]. Due to its implication in peripheral arterial occlusive disease (PAOD), a major complication of lower limb arterial calcification is amputation [4–6]. Although endovascular and open surgery are efficient procedures in diabetic PAOD, arterial calcification is a risk factor of arterial revascularization failure [7, 8]. Understanding the pathophysiology of the progression of peripheral arterial calcification is important in opening new therapeutic perspectives.

For a long time, the mechanism of arterial calcification was considered as a passive process resulting in the precipitation and deposition of hydroxyapatite crystals. But now vascular calcification is considered as a complex and actively controlled molecular process of “arterial ossification”, involving the differentiation of vascular smooth muscle cells and other cells into osteoblast-like cells [4, 9, 10]. There is growing evidence that the OPG (osteoprotegerin)/RANKL(Receptor Activator of Nuclear factor κB ligand) signaling axis could also play a central role [11, 12]. Others pathways are also suspected such as i) mechanical stress and hypertension, ii) induction of calcification by lipids, inflammation, glucose, advanced glycation end-products (AGEs) and their soluble receptor (sRAGE) [9, 13–15]. Furthermore, neuropathy could also participate in the calcification process [4].

The prospective DIACART study was designed to look at the potential determinants of lower limb arterial calcification progression in type 2 diabetes. The primary objective was to determine if the RANKL pathway is associated with the progression of lower limb arterial calcification in Type 2 Diabetes. Secondary objectives were to assess association between the others proteins of bone remodeling, traditional cardiovascular risk factors, markers of inflammation, metabolic parameters and the progression of lower limb arterial calcification.

## Methods

### Study design

The DIACART (for “DIAbète et Calcification ARTerielle”) study is a prospective monocentric cohort study [4]. The recruitment period extended from February to October 2014. Inclusion criteria were: Type 2 Diabetes with at least a history of coronary artery disease and/or peripheral arterial occlusive disease and/or age > 50 years for men and > 60 years for women. Exclusion criteria were: [1] an estimated glomerular filtration rate (GFR) calculated by the modification of diet in renal disease equation < 30 ml/min, [2] a history of lower limbs angioplasty and/or bypass, or [3] type 1 diabetes. The study was approved by the local ethics committee and registered in ClinicalTrials.gov (Identifier: NCT02431234). All patients were informed of the study objectives and procedures. Participants gave their written informed consent of participation prior to inclusion.

### Study follow-up

At the inclusion visit and after a mean follow up of 31.2 ± 3.9 months (median 30.5 months and range 26-44.8 months), all patients had a clinical evaluation, laboratory blood tests, and helicoidal CT (computerized tomography) scans. Patient interviews focused on comorbidities and their personal disease history. Their medical records were reviewed to check the clinical information and to record concomitant treatments. Ischemic cardiomyopathy was defined as a history of myocardial infarction, acute coronary syndrome or any surgical procedures undergone due to coronary artery disease.

### Assessment of peripheral neuropathy

The physician who performed the clinical tests was not informed of the calcification score nor the laboratory results. Peripheral neuropathy was assessed by the Neuropathy Disability Score (NDS) [16]. It assesses sensory vibration of the big toe using a 128-Hz tuning fork, temperature sense on the dorsum of the foot using a tuning fork with a beaker of ice or warm water, pinprick sense by applying a pin near the nail of the big toe and the Achilles reflex test. Each sensory test scores 0 for normal and 1 for abnormal sensation, on each foot. Achilles reflexes score 0 if they are present, 1 if they are present with reinforcement and 2 if they are absent, for each foot. An NDS ≥ 6 leads to the diagnosis of diabetic peripheral neuropathy.

### Laboratory evaluations

Blood and urine samples were collected after an overnight fast for the measurement of routine biochemistry diagnostic tests, glucose, glycated hemoglobin (HbA1c), cholesterol (total and HDL), triglycerides, high-sensitivity C-reactive protein (hsCRP), calcium, phosphate, urine albumin and creatinin.

Total OPG was measured by ELISA (ELISA MicroVue; Quidel Corporation), and soluble RANKL (sRANKL) concentrations were evaluated using the human RANKL Single Plex kit from Millipore (Ref: HBN51K1RANKL; eBioscienceo). With this kit, the minimum detectable concentration of sRANKL is 4.8 pg/ml and inter- and intra-assay precision is below 6% coefficient of variation. The OPG to sRANKL concentration ratio was calculated for each patient without any postanalytical modifications.

Circulating total adiponectin concentrations were measured in serum using an enzyme-linked immunosorbent assay kit (ALPCO, Eurobio, Paris, France) as recommended by the manufacturer, with the lowest detection limit of 0.4 µg/ml for total adiponectin. Inter-assay coefficients of variation of low and high human pool controls for total adiponectin were 7.93% and 8.46%, respectively.

Selected assays (including desphospho–uncarboxylated MGP (dp-ucMGP) and total-uncarboxylated MGP (t-ucMGP) assays) were performed on thawed samples, which had been frozen and stored at − 80 °C. A dual-antibody ELISA was used to measure dp-ucMGP levels; the capture antibody was directed against the non-phosphorylated MGP sequence 3–15 (mAb-dpMGP; VitaK BV, Maastricht, Netherlands) and the detecting antibody was directed against the uncarboxylated MGP sequence 35–49 (mAb-ucMGP; VitaK BV). The same antibodies have already been used elsewhere for immunohistochemical staining [15, 17]. Intra-assay variability was 5,6% for dp-ucMGP and 8,9% for t-ucMGP, while inter-assay variability was 9,9% for dp-ucMGP and 11,4% for t-ucMGP. A competitive (single-antibody) ELISA was used to measure t-ucMGP levels, as described previously [18, 19].

Soluble RAGE (sRAGE) was measured on plasma samples with a commercially available ELISA kit (Quantikine Human RAGE Immunoassay, R&D Systems, Minneapolis, USA; see http://www.rndsystems.com/Products/DRG00 for detailed description of the measurement method).

Advanced Glycation End products (Carboxymethyllysine (CML), Methylglyoxal-derived hydroimidazolone1 (MG‐H1) and pentosidine) were determined on serum samples by liquid chromatography coupled with tandem mass spectrometry (API4000 system ABSciex, Les Ulis, France) [20]. AGEs concentrations were expressed as a ratio of total protein concentrations.

Total cholesterol and triglyceride concentrations were determined by an automated enzymatic method (Konelab, Thermoclinical Labsystems, Cergy Pontoise, France and Biomerieux, Marcy L’Etoile, France, respectively). HDL-cholesterol was measured by a direct method (Konelab). LDL-cholesterol was calculated using Friedewald’s equation on triglycerides ≤ 3.9 mmol/l or directly measured when triglycerides were > 3.9 mmol/l using a Konelab kit. Inter- and intra-assay coefficients of variation were 2.2% and 0.9%, 1.3% and 0.9%, 3.5% and 0.97%, 1.3% and 0.9%, respectively for total cholesterol, HDL-cholesterol, triglycerides and LDL-cholesterol, respectively.

Serum human Fetuin A was evaluated using ELISA kit (TECOmedical Group, France) and was performed according to instructions provided by Epitope Diagnostics (intra-assay coefficient of variation: < 5.5%, inter-assay coefficient of variation: < 6.8%; detection limit of the assay: 5.0 ng/ml).

Serum human C-terminal FGF23 (Fibroblast Growth Factor 23) was determined using an ELISA kit (TECOmedical Group, France) and was performed according to the instructions provided by Immutopics (intra-assay coefficient of variation: < 2.4%, inter-assay coefficient of variation: < 4.7%; detection limit of the assay: 1.5 RU/ml).

IL-6 (Interleukine-6) was quantified using automated assays with Access 2 (Beckman Coulter Inc, Villepinte, France) according to the manufacturer’s instructions. IGF-1 (Insulin like Growth Factor 1), 25-hydroxyvitamin D, intact parathyroid hormone (iPTH) were measured by a single step chemiluminescence sandwich method on the Liaison XL (DiaSorin) analyzer. Because IGF-1 decreases with age, a standardized IGF-1 score was calculated as previously described [IGF-1 score = (log [IGF-1 (micrograms per liter)] + 0.00625 × age − 2.555)/0.104] [21].

### Assessment of insulin resistance

Insulin resistance was evaluated by the TyG index (triglycerides glucose index), which was calculated as ln(fasting triglycerides [mg/dL]*fasting glucose [mg/dL]/2) [22].

### Imaging for below-knee arterial calcification score

Below-knee artery calcification score was obtained after scanning with a 128-slice multidetector dual source CT-scanner (Somatom Definition Flash, Siemens Healthineers Healthcare, Erlangen, Germany) without contrast, from the base of the patella down to the ankle. Three millimeter cross-sectional slices were analyzed. The analysis was performed by radiologists who were blinded to the results of color duplex ultrasonography, laboratory tests or clinical examination, using a commercially available software package (Heartbeat CaScore, Philips Healthcare, Eindhoven, Netherlands). On cross-sectional images, areas of calcification along below-knee arteries with a density of ≥ 130 Hounsfield units attenuation and a surface area of > 1 mm^2^ were identified semi automatically. Calcification score, determined according to the method described by Agatston et al., was obtained separately for each of the main below-knee arteries (distal popliteal, anterior tibial, posterior tibial and peroneal arteries) and then added up to obtain the total calcification score [23]. Below-knee artery calcification scoring was performed at the inclusion visit and at the end of the study [4].

### Statistical analysis

Quantitative variables were described by their mean, standard deviation, median and quartiles Q1–Q3. Qualitative variables were described by their frequency and percentage. The effect of baseline serum RANKL on the progression of calcification of leg arteries between baseline and the end of the follow-up was analyzed using univariable and multivariable linear regression model adjusted on arterial calcification score at baseline. This method is fully equivalent to an analysis of covariance (ANCOVA). Distributions of biological parameters were checked graphically and those with a log-normal distribution were subsequently transformed before any analysis to improve normality. This model was adjusted on baseline cardiovascular risk factors and other factors known to be associated with vascular calcification (age, gender, tobacco use, hypertension, waist circumference, BMI, triglycerides, total cholesterol, HDL-cholesterol, LDL-cholesterol, ApoA1, urinary albumin-creatinine ratio, HbA1c, lower limb log calcification score at baseline, hsCRP, parathormone, glomerular filtration rate (MDRD), and duration of follow up). Cook distance was used for the detection of highly influential observations on the coefficient estimates. A backward stepwise variable selection procedure based on the Akaike Information Criterion was used to select the final multivariate model. Coefficient estimates were provided with their corresponding 95% confidence intervals.

The effects of other bone remodeling factors, markers of inflammation and glycation (Calcium, iPTH, OPG, MGP, IGF-1, Fetuin A, FGF-23, hsCRP, IL-6, carboxyméthyllysine, MG-H1, pentosidine, RAGE) were evaluated with multivariate linear regression model using the same procedure.

Significance was defined as p-values of less than 0.05. Statistical analyses were performed using R 3.5.1 (http://www.R-project.org).

### Sample size

It was assumed that the correlation between the logarithm of the serum RANKL level and the logarithm of the artery calcification score at 2 years, adjusted on the covariates, is about 0.20 (or an R^2^ of 0.04). In addition, it was assumed that the multivariate linear model (based on RANKL and associated risk factors) will explain about 15% of the variability. Under these assumptions, at least 169 patients were needed to demonstrate a significant effect of RANKL on the calcification score with an alpha risk of 5% (bilateral formulation) and a power of 80%. In order to take into account 15% of patients lost to follow-up, 198 subjects were planned for the study.

## Results

At baseline, a total of 198 patients were included in the DIACART study. During the study follow-up (mean 31.2 ± 3.9 months; median 30.5 months and range 26–44.8), 18 patients were lost to follow up and 11 patients died before the second determination of calcification score. RANKL measurements were missing in 6 patients. This led to 163 evaluable patients.

### Patients’ characteristics

The characteristics of the participating patients at baseline (n = 163) are presented in Table 1. At baseline, patients were predominantly middle aged (median [Q1–Q3], 65 years [58–70]) overweight (median [Q1–Q3] BMI, 28.39 kg/m^2^ [25.44;31.77]) male (80%), with a long duration of diabetes (median [Q1–Q3], 12 years [6–20]), and a relatively good glycemic control (median [Q1–Q3] HbA1c, 7.4% [6.9;8.25]; 57 mmol/mol [52;67]). Less than 20% of the patients had laserized retinopathy or neuropathy. Nearly a third of the patients had nephropathy but the GFR (median [Q1–Q3], 77 ml/min [63.5;89]) was relatively preserved as expected since a GFR of < 30 ml/min was an exclusion criterion. Most of the patients had ischemic cardiomyopathy (69%), were smokers (60%) and treated for hypertension (80%) with arterial pressure at target (126 ± 17 mmHg). They often took statins (90%), antiplatelet therapies (83%), β-blockers (63%) and renin-angiotensin system inhibitors (79%). Diabetes was often treated with metformin, insulin and sulfonylurea (respectively 79%, 57% and 46%).

**Table 1:** Baseline characteristics of the patients (n = 163)

Age (years)	65 [58–70]
Sex male n (%)	130 (80)
BMI (kg/m^2^)	28.39 [25.44;31.77]
Waist circumference (cm)	101 [93;110]
Smoking habit (active or past) n (%)	97(60)
Diabetes duration (years)	12 [6–20]
Glycemia (mmol/l)	7.7 [6.2;9.25]
HbA1c % (mmol/mol)	7.4 [6.9;8.25] (57 [52;67])
Ischemic cardiomyopathy n (%)	117 (69)
Calcification score	526.73 [55.25–2253.41]
NDS ≥ 6 n (%)	25 (15)
Laserised retinopathy n (%)	26 (16)
eGFR MDRD (ml/min)	77 [63.5;89]
Microalbuminuria (mg/l)	20 [8.55;66.95]
urinary albumin-creatinine ratio > 3 mg/mmol n (%)	53 (33)
Hypertension n (%)	131 (80)
Triglycerides (mmol/l)	1.25 [0.91;2]
Total Cholesterol (mmol/l)	3.52 [2.7;4.31]
HDL Cholesterol (mmol/l)	1.06 [0.88;1.27]
LDL Cholesterol (mmol/l)	1.84 [1.46;2.35]
SBP (mmHg)	124 [115;135]
DBP (mmHg)	72 [67;78]
hsCRP (mg/l)	1.20 [0.5;2.6]
IL-6 (pg/ml)	2.80 [2.1;4]
Adiponectine	3.50 [2.7;5.05]
TyG index	8.99 [8.53;9.49]
IGF-1 (ng/ml)	139 [105;170]
Standardized IGF-1 (ng/ml)	− 0.12 (− 1.21 to 0.8)
MG‐H1 (µmol/g prot)	2.79 [2.48;3.06]
Pentosidine (nmol/g prot)	1.10 [0.84;1.42]
Carboxyméthyllysine (µmol/g prot)	0.14 [0.13;0.16]
sRAGE (pg/ml)	828.77 [582.61;1181.96]
Corrected calcium (mmol/l)	2.31 [2.24;2.38]
Phosphorus (mmol/l)	1.01 [0.91;1.13]
iPTH (pg/ml)	47.05 [36.75;64.97]
sRankl (pmol/l)	8.06 [2.59;16.8]
OPG (pmol/l)	6.06 [5.26;7.13]
OPG/RANKL	0.73 [0.33–2.25]
Fetuin A (g/l)	0.65 [0.51;1.13]
FGF-23 (UI/ml)	20.87 [11.65;34.07]
t-ucMGP	4733 [3680–5490]
Dp-ucMGP	561 [339–761]
Insulin use n (%)	75 (46)
Metformin use n (%)	130 (80)
Sulfonylurea use n (%)	71 (43)
Statin use n (%)	146 (90)
Antiplatelet use n (%)	136 (83)
ARB and ACE inhibitors use n (%)	128 (79)
Beta-blocker use n (%)	102 (63)

Data are given as median (and quartiles Q1–Q3) or as the number (and percentage) for binary variables

*ARB* angiotensin receptor blockers, *ACE* angiotensin converting enzyme, *BMI* body mass index, *CVD* cardiovascular disease, *DBP* diastolic blood pressure, *FGF-23* fibroblast growth factor 23, *eGFR MDRD* estimated glomerular filtration rate calculated with the modification of diet in renal disease formula, *HbA1c* haemoglobin A1C, *HDL* high density lipoprotein, *hsCRP* high sensitivity C-reactive protein, *IGF-1* insulin growth factor-1, *IL-6* interleukin 6, *iPTH* intact parathyroid hormone, *LDL* low density lipoprotein, *MG‐H1* Methylglyoxal-derived hydroimidazolone1, *dp-ucMGP* desphospho-uncarboxylated matrix gla protein, *t-ucMGP* Total uncarboxylated matrix gla protein, *NDS* neuropathy disability score, *OPG* osteoprotegerin, *SBP* systolic blood pressure, *sRAGE* soluble form of receptor for advanced glycation end products, *sRANKL* soluble form of Receptor Activator of Nuclear factor kB Ligand, *TyG* triglyceride glucose index

### Progression of lower limb calcification and independent predictors

At baseline, mean ± SD and median lower limb arterial calcification scores were 2364 ± 5613 and 527 respectively. At the end of the study, mean ± SD and median arterial lower limb calcification scores were 3739 ± 6886 and 1355 respectively. In Table 2, lower limb arterial calcification scores are shown for all patients and according to circulating RANKL quartiles at baseline.

**Table 2:** Calcium score at baseline and at the end of the follow-up in all the population and according to circulating RANKL quartiles (n = 163)

	All	sRANKL quartiles			
		< Q1	[Q1–Q2]	[Q2–Q3]	> Q3
Calcium score at baseline	527 [55–2253]	1200 [152–2311]	535 [66–1339]	384 [237–3175]	815 [55–4488]
Calcium score at the end of follow-up	1355 [167–4235]	2204 [410–4084]	1355 [133-3393]	1234 [404–5701]	1840 [153-7265]

Data are given as median (and quartiles Q1–Q3). sRANKL soluble form of Receptor Activator of Nuclear factor kB Ligand

In univariate analysis (cf Table 3), lower limb log calcification score at the end of follow-up is correlated with (β coefficient [slope], 95% CI, p-value) baseline log calcification score (0.88, 0.81–0.95, p < 0.001), age (0.15, 0.06–0.25, p < 0.001), male sex (3.51, 1.64–5.38, p < 0.001), tobacco use (for patients without tobacco consumption, − 1.69; − 3.27 to − 0.12, p = 0.0035), ischemic cardiomyopathy (3.73, 2.15–5.32, p < 0.001), MDRD (− 0.06, − 0.10 to − 0.02, p = 0.005), t-ucMGP (− 2.74, − 4.85 to − 0.64, p = 0.011), dp-ucMGP (1.61, 0.53–2.70, p = 0.004), Fetuin A (− 1.82, − 3.36 to − 0.29, p = 0.02), pentosidine (2.16, 0.35–3.96, p = 0.02), CML (1.61, 0.53–2.70, p = 0.004), MG-H1 (− 3.15, − 5.79 to − 0.51, p = 0.020) and iPTH (0.04, 0.01–0.07, p = 0.012). But peripheral neuropathy (defined by NDS ≥ 6) was not associated with lower limb arterial calcification progression (0.15, − 0.86–1.17, p = 0.765). HbA1c, diabetes duration, calcemia, phosphoremia, RAGE, Fetuin A, FGF-23, IGF-1, LDL-cholesterol, HDL-cholesterol, total cholesterol, blood pressure, BMI, hsCRP and IL-6 were also not associated with lower limb arterial calcification progression.

**Table 3:** Univariate linear regression analysis: variables associated with calcification score at the end of follow-up (n = 163 patients; mean follow-up duration = 31.20 ± 3.86 months)

	β coefficient [slope]	IC 95	p value
Log(calcium score at baseline)	0.88	[0.81;0.95]	*< 0.001*
Follow-up duration	− 0.07	[− 0.27;0.13]	0.49
Age	0.15	[0.06;0.25]	*0.001*
Sex (male)	3.51	[1.64–5.38]	*< 0.001*
Smoking habits (never)	− 1.69	[− 3.27 to − 0.12]	*0.03*
Hypertension (absence)	− 0.75	[− 2.72 to 1.22]	0.45
BMI	− 0.05	[− 0.2 to 0.09]	0.48
Waist circumference	0	[− 0.02 to0.03]	0.75
HbA1c	0.05	[− 0.53 to 0.62]	0.87
Diabetes duration	0.07	[− 0.01;0.14]	0.09
NDS ≥ 6	0.15	[− 0.86 to 1.17]	0.76
Ischemic cardiomyopathy	3.73	[2.15–5.32]	*< 0.001*
eGFR MDRD	− 0.06	[− 0.01; − 0.02]	*0.005*
hsCRP	− 0.16	[− 0.47 to 0.16]	0.32
Log IL-6	1.05	[− 0.22 to 2.32]	0.10
Triglycerides	0.43	[− 0.3 to 1.16]	0.25
Cholesterol total	− 0.51	[− 1.39 to 0.37]	0.25
HDL	− 2.01	[− 4.27 to 0.25]	0.08
LDL	0.56	[− 1.6 to 0.48]	0.29
Log(sRANKL)	0.04	[− 0.39 to 0.46]	0.86
Log(OPG)	1.84	[− 1.14 to 4.83]	0.22
Log(OPG/sRANKL)	0	[− 0.41 to 0.41]	0.99
iPTH	0.04	[0.01–0.07]	*0.01*
Log (Corrected calcium)	− 9.99	[− 26 to 6.02]	0.22
Log(t-ucMGP)	− 2.74	[− 4.85 to − 0.64]	*0.01*
Log(dp-ucMGP)	1.61	[0.53–2.7]	*0.004*
Log(FGF-23)	0.56	[− 0.03 to 1.16]	0.06
Log(Fetuine A)	− 1.82	[− 3.36 to − 0.29]	*0.02*
Log(Pentosidine)	2.16	[0.35-3.96]	*0.02*
Log(Carboxymethylysine)	1.30	[− 2.67 to 5.27]	0.52
Log(MG-H1)	− 3.15	[− 5.79 to − 0.51]	*0.02*
Log(sRAGE)	0.69	[− 0.79 to 2.16]	0.36
Log(IGF-1)	− 2.06	[− 4.15 to 0.02]	0.05
Standardized IGF-1	− 0.24	[− 0.75 to 0.27]	0.36
Log adiponectin	1.59	[0.12–3.06]	0.03
TyG INDEX	0.02	[− 0.02 to 0.06]	0.25
Statin use	1.62	[− 0.93;4.17]	0.21

*BMI* body mass index, *DBP* diastolic blood pressure, *FGF-23* fibroblast growth factor 23, *eGFR MDRD* estimated glomerular filtration rate calculated with the modification of diet in renal disease formula, *HbA1c* haemoglobin A1C, *hsCRP* high sensitivity C-reactive protein, *IGF-1* insulin growth factor-1, *IL-6* interleukin 6, *iPTH* intact parathyroid hormone, *MG‐H1* Methylglyoxal-derived hydroimidazolone1, *dp-ucMGP* desphospho-uncarboxylated matrix gla protein, *t-ucMGP* Total uncarboxylated matrix gla protein, *NDS* neuropathy disability score, *OPG* osteoprotegerin, *sRAGE* soluble form of receptor for advanced glycation end products, *sRANKL* soluble form of Receptor Activator of Nuclear factor kB Ligand, *TyG* triglyceride glucose index

p-values indicating significance are shown in italics type

In multivariate analysis (cf Table 4), and after exclusion of highly influential observations, progression of lower limb arterial calcification was associated with (β coefficient [slope], 95% CI, p-value) baseline log calcification score (1.02, 1.00–1.04, p < 0.001), duration of follow up (0.04, 0.01–0.06, p = 0.004), ischemic cardiomyopathy (0.36, 0.15–0.57, p = 0.001), statin use (0.39, 0.06–0.72, p = 0.023), triglycerides (0.11, 0.03–0.20, p = 0.007) and log RANKL (0.07, 0.02–0.11, p = 0.016). Similarly, the analysis of the predictive power of the others biomarkers showed that Log(OPG/RANKL ratio) was also associated with lower limb arterial calcification progression (− 0.06, − 0.12–0, p = 0.03) while OPG alone (− 0.15, − 0.52–0.23, p = 0.409) was not. Other markers potentially involved in arterial calcification pathophysiology were not implicated in lower limb arterial calcification progression in the DIACART study.

**Table 4:** Final multivariate linear regression model: variables independently associated with the progression of calcification score during follow-up (n = 163 patients; mean follow up duration = 31.2 ± 3.86 months)

	β	IC95	p value
Log(calcium score at baseline)	1.02	[1–1.04]	*< 0.001*
Follow-up duration	0.04	[0.01; 0.06]	*0.004*
Ischemic cardiomyopathy	0.36	[0.15; 0.57]	*0.001*
Log(sRANKL)	0.07	[0.02; 0.11]	*0.016*
Log(OPG/sRANKL)	− 0.06	[− 0.12–0]	*0.03*
Triglycerides	0.11	[0.03; 0.2]	*0.007*
Statin use	0.39	[0.06; 0.72]	*0.023*

*OPG* osteoprotegerin, *sRANKL* soluble form of Receptor Activator of Nuclear factor kB Ligand

p-values indicating significance are shown in italics type

## Discussion

Our study confirms that the prevalence of arterial calcification in the lower limbs of patients with type 2 diabetes and with a high cardio-vascular risk is high, but also shows that this pathological process progresses significantly. The association of the calcification progression with the severity of pre-existing calcification, the duration of follow-up and a history of ischemic cardiomyopathy has already been described. But our results highlight the putative role of the RANK/RANKL pathway and circulating triglycerides in this phenomenon.

### RANKL/RANK/OPG system and lower limb arterial calcification

Our results suggest that an emerging pathway in arterial calcification pathophysiology is the RANKL/RANK/OPG system. RANKL, which is produced by osteoblasts, vascular cells, stromal cells, T-cells, macrophages and monocytes, binds to its receptor RANK which is expressed by osteoclasts but also by vascular smooth muscular cells (VSMCs). This binding activates several downstream targets such as the Nuclear Factor-kappa B (NFkB). In contrast, OPG acts as a decoy receptor blocking the RANKL-RANK interaction [24]. RANKL actively promotes vascular calcification by inducing, via its receptor RANK, differentiation of VSMCs into osteoblast-like cells [12, 25]. RANK is a member of the Tumor Necrosis Factor Receptor (TNFR) superfamiliy, and TNFR- 1 is associated with the risk of major amputation in diabetes [26]. OPG was shown to be associated with carotid intima-media thickness in T2D patients [27] and is also suspected to be involved in the regulation of the vascular calcification process. Indeed, OPG-deficient mice develop accelerated arterial calcification whereas inactivation of RANKL signaling in these mice counteracts this effect [28]. This suggests that RANKL plays a central role in the regulation of vascular calcification, whereas OPG would rather play a modulatory function. In human, both tissue and serum RANKL have been associated with lower limb artery and carotid calcification [12, 29]. The RANKL/OPG ratio has also been correlated with the coronary artery calcium score [30]. In the DIACART study, both circulating RANKL and RANKL/OPG ratio, but not serum OPG, were correlated with the progression of lower limb arterial calcification. Thus when circulating RANKL concentration increases, it would be associated with an acceleration of the arterial calcification process.

### Lipids and lower limb arterial calcification

We also found and association between plasma triglycerides and the progression of lower limb arterial calcification. However we did not find any correlation of the calcification progression with others lipids, although sortilin, which is implicated in lipid metabolism, extra-cellular matrix mineralization and atherosclerotic plaque burden, was recently found to be associated with the presence of PAOD in T2D [31]. Previous studies have shown a relationship between triglycerides and coronary artery calcium score progression in patients with or without diabetes [32, 33]. When accumulated in the tissue, triglycerides can generate species such as free fatty acids, sphingolipids and particularly ceramides, which could induce pathophysiological pathways involved in vascular calcification. Saturated fatty acids are associated with vascular disease and with the development of vascular smooth muscle cell calcification via the NF-kB pathway induction [34]. Among sphingolipids species, ceramides are known to induce human vascular smooth muscle cell calcification via p38 mitogen-activated protein kinase signaling [35]. It would be interesting to study the role of lipid derivatives, such as sphingolipids, on lower limb vascular calcification development occurring in type 2 diabetes.

### Insulin resistance and lower limb arterial calcification

Circulating triglycerides are also a strong marker of insulin resistance and visceral obesity. However in our study others biomarkers of insulin resistance and/or visceral obesity (Triglyceride-Glucose index (TyG index), waist circumference and serum adiponectin) did not correlate with the progression of lower limb vascular calcification. This suggests that triglycerides act through an independent pathway to insulin resistance and visceral obesity.

### Inflammation and lower limb arterial calcification

Recently low levels of Omentin-1, which is an adipokine with anti-inflammatory properties, were shown to be associated with the presence and severity of PAOD in T2D [36]. However inflammatory markers like IL-6 and hsCRP were not associated with the progression of the calcification in our study.

### Therapeutic perspectives

Denosumab is a human monoclonal antibody which inhibits the RANKL pathway. It is one of the latest therapeutic options for osteoporosis [37]. It has been shown that Denosumab attenuates aortic calcification in a murine model [38]. However, in the human, a study has prospectively explored the effects of 12 months of Denosumab treatment on coronary artery calcium scores in 48 patients on hemodialysis [39]. Interestingly, the coronary artery calcium score of patients treated with Denosumab did not increase, even though these patients had very high coronary artery calcium scores at baseline. These data suggest that Denosumab could at least stop the progression of vascular calcification in patients highly exposed to this risk. But interventional studies are needed to determine if targeting RANKL may be beneficial in peripheral arterial calcification progression in type 2 diabetes.

To our knowledge, the effect of fibrates on arterial calcification has not been explored. Hence, targeting triglycerides could be another interesting therapeutic path to take.

As previously reported with accelerated coronary artery calcification in T2D patients with advanced atherosclerosis, statin use is associated with the progression of lower limb arterial calcification in our study [40]. Statins are thought to increase the density of arterial calcification to improve plaque stability but don’t seem able to reduce lower limb arterial calcification progression.

The strengths of the present study are its prospective design, the objective quantitative assessment of arterial calcification by CT, the measurement of numerous original markers and the use of a high sensitive kit to measure RANKL. The limitations are the relative small sample size, the absence of a non-diabetic control population, and the absence of distinction between intima and medial calcification [41]. In addition, uncalcified plaques were not assessed in this study.

## Conclusions

Lower limb arterial calcification, which has serious clinical consequences, is a frequent pathological process in patients with type 2 diabetes and high cardiovascular risk. It progresses rapidly. The demonstration of the association of RANKL and triglycerides with this progression open up new therapeutic perspectives. Further interventional studies are therefore required to address these issues fully.

## Data Availability

Data are available on request from the corresponding author.

## Abbreviations

AGEs: Advanced glycation end-products
BMI: Body Mass Index
CML: Carboxymethyllysine
DIACART: Acronym of « DIAbète et Calcification ARTerielle »
dp-ucMGP: Desphospho–uncarboxylated Matrix Gla Protein
GFR: Glomerular filtration rate
HbA1c: Glycated hemoglobin
hsCRP: High-sensitivity C-reactive protein
IGF-1: Insulin like growth factor 1
IL-6: Intelukine-6
iPTH: intact parathyroid hormone
MG‐H1: Methylglyoxal-derived hydroimidazolone1
NDS: Neuropathy disability score
OPG: Osteoprotegerin
PAOD: Peripheral arterial occlusive disease
RANK: Receptor Activator of Nuclear factor κB
RANKL: Receptor Activator of Nuclear factor κB Ligand
sRAGE: Soluble receptor of advanced glycation end-products
sRANKL: Soluble RANKL
t-ucMGP: Total-uncarboxylated Matrix Gla Protein
TyG index: Triglycerides glucose index
